# Adipocyte-Specific Laminin Alpha 4 Deletion Preserves Adipose Tissue Health despite Increasing Adiposity

**DOI:** 10.3390/biomedicines10092077

**Published:** 2022-08-25

**Authors:** Jennifer L. Bailey, David H. Burk, Susan J. Burke, Scott D. Reed, Sujoy Ghosh, Carrie M. Elks

**Affiliations:** 1 Matrix Biology Laboratory, Pennington Biomedical Research Center, Baton Rouge, LA 70808, USA; 2 Cell Biology and Bioimaging Core, Pennington Biomedical Research Center, Baton Rouge, LA 70808, USA; 3 Immunogenetics Laboratory, Pennington Biomedical Research Center, Baton Rouge, LA 70808, USA; 4 North American Science Associates, Northwood, OH 43619, USA; 5 Cardiovascular and Metabolic Disease Program and Center for Computational Biology, Duke-NUS Graduate Medical School, Singapore 169857, Singapore; 6 Laboratory of Computational Biology, Pennington Biomedical Research Center, Baton Rouge, LA 70808, USA

**Keywords:** adipocyte, adipose tissue, extracellular matrix, laminin alpha 4, obesity, basement membrane

## Abstract

Laminins are heterotrimeric glycoproteins with structural and functional roles in basement membranes. The predominant laminin alpha chain found in adipocyte basement membranes is laminin α4 (LAMA4). Global LAMA4 deletion in mice leads to reduced adiposity and increased energy expenditure, but also results in vascular defects that complicate the interpretation of metabolic data. Here, we describe the generation and initial phenotypic analysis of an adipocyte-specific LAMA4 knockout mouse (*Lama4*^AKO^). We first performed an in-silico analysis to determine the degree to which laminin α4 was expressed in human and murine adipocytes. Next, male *Lama4*^AKO^ and control mice were fed chow or high-fat diets and glucose tolerance was assessed along with serum insulin and leptin levels. Adipocyte area was measured in both epididymal and inguinal white adipose tissue (eWAT and iWAT, respectively), and eWAT was used for RNA-sequencing. We found that laminin α4 was highly expressed in human and murine adipocytes. Further, chow-fed *Lama4*^AKO^ mice are like control mice in terms of body weight, body composition, and glucose tolerance, although they have larger eWAT adipocytes and lower insulin levels. High-fat-fed *Lama4*^AKO^ mice are fatter and more glucose tolerant when compared to control mice. Transcriptionally, the eWAT of high-fat fed *Lama4*^AKO^ mice resembles that of chow-fed control mice. We conclude from these findings that adipocyte-specific LAMA4 deletion is protective in an obesogenic environment, even though overall adiposity is increased.

## 1. Introduction

Adipocytes, or fat cells, are each surrounded by a thin sheet of extracellular proteins known as the basement membrane, which often contains type IV collagen, nidogen (entactin), perlecan, and laminins [1,2,3]. Basement membranes perform several critical functions; they provide mechanical stability and participate in cell differentiation, survival, and migration. Laminins (LN) are glycoproteins composed of heterotrimers of α, β, and γ chains and are named according to their chain compositions (for example, LN-411 is comprised of the α4, β1, and γ1 chains) [2,3]. In addition to their structural roles, laminins also interact with surface receptors on neighboring cells to participate in cell signaling. The LN α4 chain (LAMA4) is the alpha component of LN-411 and LN-421 and is the major alpha chain found in the adipocyte basement membrane [4,5]; it is also widely distributed in the peripheral nerves, heart, and vascular endothelial basement membranes [2,6,7,8].

The physiological function of LAMA4 has been assessed in mice with global *Lama4* deletion (*Lama4^−/−^*), originally generated by the Tryggvason laboratory [9]. Adult *Lama4^−^*^/−^ mice exhibit extensive disorganization of microvessels, and newborns exhibit subcutaneous and muscular hemorrhages and anemia along with delayed capillary basement membrane deposition [9]. Approximately 80% of *Lama4^−/−^* mice survive past one week after birth; these mice grossly resemble control littermates but exhibit cardiac hypertrophy and ischemia with increased sudden death [10]. Data from these studies have revealed critical roles for LAMA4 in microvessel growth, endothelial basement membrane strength and integrity, and cardiac function.

The metabolic health of male *Lama4^−/−^* mice has been extensively profiled [4,11,12]. These animals gain less weight in response to a high-fat diet (HFD) and have decreases in epididymal white adipose tissue (eWAT) mass and lipogenesis, with no comparable changes in subcutaneous AT [12]. In addition to their improved insulin sensitivity, *Lama4^−/−^* mice also have significantly higher energy expenditure, attributable to increased beiging of subcutaneous AT [4]. Most recently, adipose tissue LAMA4 levels were found to be increased in HFD-fed C57BL/6J mice and adipose tissue of people with obesity [11]. Interestingly, these LAMA4 levels did not change with weight loss. In vitro, adipocytes cultured on LN411-coated plates demonstrated alterations in genes involved in lipolysis and β-oxidation [11].

Data from studies on *Lama4^−/−^* mice have advanced our understanding of adipose tissue LAMA4 and its regulation in obesity. However, the cardiovascular derangements present in these mice complicate the interpretation of adipose tissue-specific effects of LAMA4 loss on metabolic parameters in these animals. Therefore, we hypothesized that adipocyte-specific loss of LAMA4 would result in metabolic effects that contrast with those in *Lama4^−/−^* mice.

To separate the adipose tissue-specific effects of *Lama4* loss from the cardiovascular derangements, we generated an adipocyte-specific LAMA4 knockout mouse (*Lama4*^AKO^). Our findings indicate that *Lama4*^AKO^ mice have increased adipocyte area in eWAT when fed a chow diet. When challenged with HFD, *Lama4*^AKO^ mice accrue significantly more fat mass via inguinal adipose tissue (iWAT) hypertrophy but remain glucose-tolerant. Transcriptomic analyses of eWAT suggest a less inflammatory, lipid storage-promoting environment in HFD-fed *Lama4*^AKO^ mice. These data indicate that the adipocyte-specific loss of LAMA4 is protective in the setting of diet-induced obesity and highlight the need for further studies to determine the molecular basis for this apparent protection.

## 2. Experimental Section

### 2.1. In Silico Analysis of LAMA4 or Lama4 Expression in Human or Mouse White Adipose Tissue and Adipocytes

Using the Single Cell Portal website (www.singlecell.broadinstitute.org; accessed on 15 April 2022), we explored single nucleus RNA-sequencing (RNA-Seq) data from human and mouse adipose tissue described in [13] (Study SCP1376 on Single Cell Portal). We used the “explore” function to examine *LAMA4* or *Lama4* expression in various cell types of human or mouse white AT (WAT) depots, respectively, with the clustering option set to human or mouse “WAT”. Using the same procedure, we also examined adipocyte-specific *LAMA4* or *Lama4* expression, with the clustering option set to human or mouse “adipocytes”.

### 2.2. Generation of Lama4 loxP/loxP Mice

**Figure 1:**
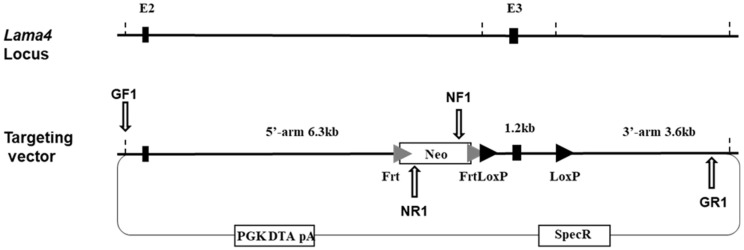
Genetic strategy and targeting vector for generation of *Lama4* loxP/loxP mice. White arrows indicate sites of primers used to determine correct homologous recombination in embryonic stem cell clones surviving positive/negative selection (GF & GR = genomic forward and reverse, respectively; NF and NR = Neo forward and reverse, respectively). Primer sequences appear in Appendix A.

Starting with the EUCOMM Lama4 targeting vector (PG00087_Y_3_B09), the Frt-LacZ-LoxP-neo-FrtLoxP portion of the vector was replaced by recombineering with an Frt-Neo-FrtLoxP element, resulting in exon 3 of the *Lama4* gene being flanked by LoxP sites. Homologous recombination was performed using the C57BL/6N-PRX-B6N #1 embryonic stem (ES) cell line (Jackson Laboratories catalog number 012448C01). Correct homologous recombination in ES cell clones surviving positive/negative selection was verified with Fidelity PCR at both flanks using primers as indicated below (Figure 1—white arrows; sequences appear in Appendix A); the presence and the correct sequence of both loxP sites were confirmed by Sanger sequencing. Targeted ES cells were injected into female albino C57BL/6J-Tyrc-2J blastocysts. Chimeric animals were mated to C57BL/6J mice to generate heterozygous offspring on the C57BL/6J genetic background. 

### 2.3. Animals and Diets

Male littermate laminin alpha 4 loxP/loxP (“floxed” mice; *Lama4*^fl/fl^) and adipocyte-specific laminin alpha 4 knockout (*Lama4*^AKO^) mice were used in these studies. Briefly, *Lama4*^fl/fl^ mice were generated by the Pennington Biomedical Research Center (PBRC) Transgenics Core as described in Section 2.1 and crossed to adiponectin-Cre mice from our in-house colony [14] (Jackson Laboratories Stock #010803) to create the adipocyte-specific LAMA4 knockout mouse (*Lama4*^AKO^). Mice were housed in a temperature-controlled (22 °C ± 2 °C) and humidity-controlled (45–55%) room under a 12-h light-dark cycle and were allowed *ad libitum* access to food and water. Mice were fed a high-fat diet (HFD; D12451; 45% fat; Research Diets) or a breeder chow diet (Purina LabDiet #5015; LabDiet, St. Louis, MO, USA) as indicated for 20 weeks. All mice were weighed weekly and body composition was measured every other week. After humane euthanasia, tissues were collected for histology, microscopy, and gene and protein expression analyses. Mice were 24–26 weeks old when tissues were collected. The Pennington Biomedical Research Center Institutional Animal Care and Use Committee approved all studies (protocols 1025 & PB21-001).

### 2.4. Validation of Cre-Mediated Adipocyte-Specific Lama4 Deletion

Total RNA was extracted from tissues (adipose tissue, heart, spleen, liver) of chow-fed *Lama4*^fl/fl^ and *Lama4*^AKO^ mice, concentrations quantified, and reverse transcription performed as previously described [15,16]. Quantitative PCR was performed and *Lama4* expression normalized to *Ppia* expression (peptidyl prolyl isomerase A). Primer sequences (Integrated DNA Technologies) for *Lama4* are as follows: TTCCTTCTCAACCAGCATACC (forward), AACAACTCGGAGAACACACTG (reverse). *Ppia* primer sequences are as follows: CCACTGTCGCTTTTCGCCGC (forward), TGCAAACAGCTCGAAGGAGACGC (reverse).

### 2.5. Intraperitoneal Glucose Tolerance Testing and Measurement of Serum Insulin and Leptin

Intraperitoneal glucose tolerance tests were performed after a 4-h fasting period using i.p. injections of 20% dextrose. Blood glucose was measured at baseline (time 0) before the administration of glucose and at the indicated post-injection time points as described previously [14]. We measured serum insulin and leptin using a mouse insulin ELISA kit from Mercodia (Uppsala, Sweden) and a Mouse/Rat Leptin Quantikine ELISA kit from R&D (Minneapolis, MN, USA), respectively, according to the manufacturer’s instructions.

### 2.6. Histologic Evaluation of Tissues and Adipocyte Size Analysis

Each animal’s liver, eWAT, brown adipose tissue (BAT), and iWAT were formalin-fixed, paraffin-embedded, and sectioned at a thickness of 5 μm. Sections were placed on slides and stained with hematoxylin and eosin (H&E) for histologic evaluation. A board-certified veterinary pathologist (S.D.R.) blinded to animal genotypes conducted a histologic evaluation of liver and eWAT sections.

Average adipocyte area (μm^2^) was determined as previously described [14]. Briefly, H&E-stained eWAT and iWAT sections were imaged using a Hamamatsu NanoZoomer digital slide scanner at 20× resolution. A custom application within VIS software version 5.0.5 (Visiopharm Corporation, Westminster, CO, USA) was used to analyze and quantify the resulting images. The number of adipocytes measured in tissues from mice on the chow diet ranged from 1975 to 9810 while adipocytes measured from mice on HFD ranged from 2370 to 12,028.

### 2.7. RNA-Seq and Bioinformatics Analysis

RNA was isolated from eWAT as described previously [14,16,17]. RNA quality was confirmed using a Bioanalyzer RNA 6000 chip (Agilent Technologies, Santa Clara, CA, USA); RNA integrity numbers were verified for all samples and ranged from 8–10. Sequencing libraries were constructed, library sizes verified, and sequencing conducted as previously described [17]. Primary data analysis was performed using the Lexogen QuantSeq pipeline V1.8.8 (Lexogen, Inc., Greenland, NH, USA) on the Bluebee platform for quality control, mapping, and read count tables. The raw and processed data are deposited in the GEO database (accession number GEO: GSE179138).

Raw count matrices of RNA sequencing data were obtained via the Rsubread [18] package in R and further processed for gene quantification and identification of differentially expressed genes using the limma package [19]. Gene counts were log2 transformed and normalized for sequencing depth via the trimmed means of M values (TMM) method [20]. The TMM-normalized data was used to identify sample outliers via Principal Components Analysis (Supplementary Figure S1). After outlier removal, genes with at least one count per million (CPM) reads in three or more samples were retained for further analysis, resulting in 14,855 genes. The mea-–variance relationship of gene-wise standard deviation to average log CPM gene signal was assessed via the ‘voom’ method [21] to generate precision weights for each observation. We subsequently utilized log CPM values and associated precision weights to generate empirical Bayes moderated t-statistics estimates for the identification of differentially expressed genes. To adjust for multiple testing, adjusted *p*-values were calculated via the false discovery rate (FDR) method [22]. Genes with absolute fold-change ≥1.5-fold, nominal *p*-value ≤ 0.001, and maximum average group expression ≥2 CPM were considered differentially expressed.

Pathway enrichment analysis on differentially expressed genes was conducted via gene-set enrichment analysis (GSEA) [23], using a custom pathway database consisting of KEGG pathways (obtained from MSigDB [23]) and several user-defined custom gene-sets. GSEA was run in the pre-ranked mode with genes ranked by their log fold-change between comparisons. Analysis was restricted to pathways containing between 15 and 250 genes, and the enrichment statistic was computed by the ‘classic’ method. Pathways with an adjusted *p*-value ≤ 0.05 were considered significantly regulated.

### 2.8. Statistical Analysis

All data are expressed as mean ± SEM. GraphPad Prism 9.0 (GraphPad Software, San Diego, CA, USA) was used for statistical analyses. For comparisons between two independent groups, a Student’s *t*-test was used and *p* < 0.05 was considered statistically significant. All sample sizes and *p*-values are listed in the figure legends.

## 3. Results

### 3.1. LAMA4 Is Highly Expressed in the Adipocyte Compartments of Human and Mouse Adipose Tissue

**Figure 2:**
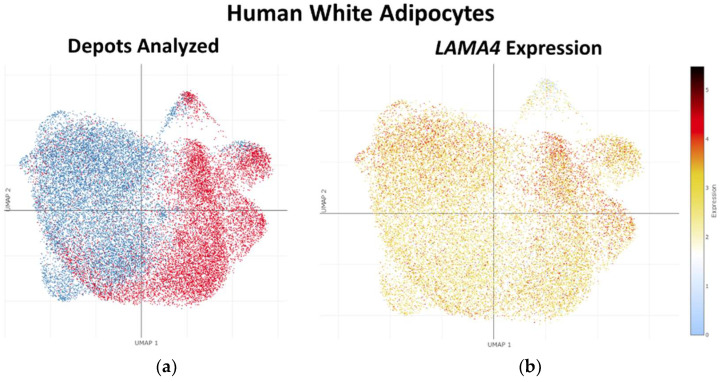

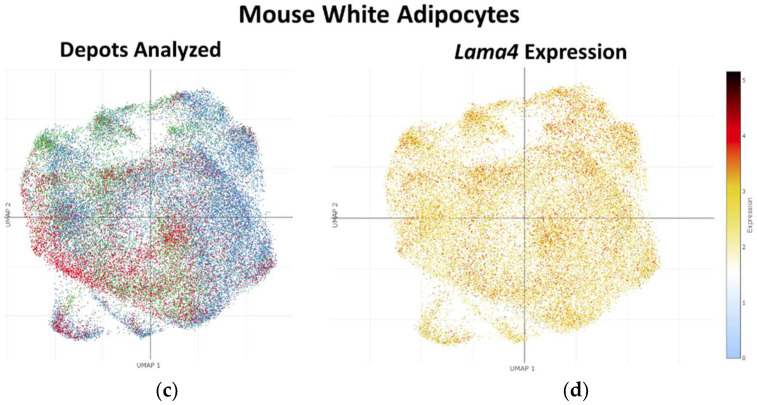
Laminin α4 is highly expressed in human and mouse adipocytes. (**a**) Uniform manifold approximation and projection (UMAP) embedding coordinates from single nucleus RNA-sequencing data for adipocytes from human WAT, red = omental depot (11,475 nuclei depicted) and blue = subcutaneous depot (14,396 nuclei depicted). (**b**) *LAMA4* expression overlaid on the UMAP coordinates from (**a**). (**c**) UMAP embedding coordinates for adipocytes from mouse WAT, red = epididymal depot (7620 nuclei depicted), blue = inguinal depot (21,921 nuclei depicted), and green = periovarian depot (10,393 nuclei depicted). (**d**) *LAMA4* expression overlaid on the UMAP coordinates from (**c**). Data used are from [13].

An in-silico analysis of a large adipose tissue single cell/single nucleus RNA-sequencing dataset [13] revealed that, in humans, *LAMA4* is expressed in several immune and non-immune cell populations of WAT, with the highest expression appearing in adipocytes and mesothelial cells (Supplementary Figure S2). In mice, *Lama4* expression is restricted to non-immune cells, with the highest expression noted in adipocytes (Supplementary Figure S3). A closer examination of the adipocyte subclusters from this dataset revealed that *LAMA4* and *Lama4* are evenly expressed between visceral and subcutaneous depots in both humans (Figure 2a,b) and mice (Figure 2c,d), respectively.

### 3.2. Body Mass and Body Composition of Chow-Fed Lama4^fl/fl^ and Lama4^AKO^ Mice

**Figure 3:**
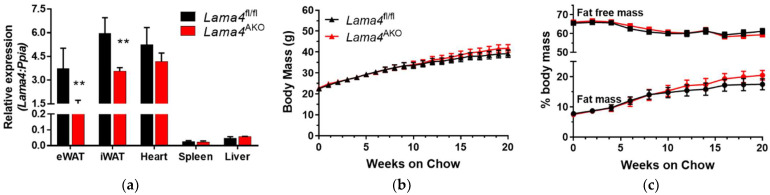

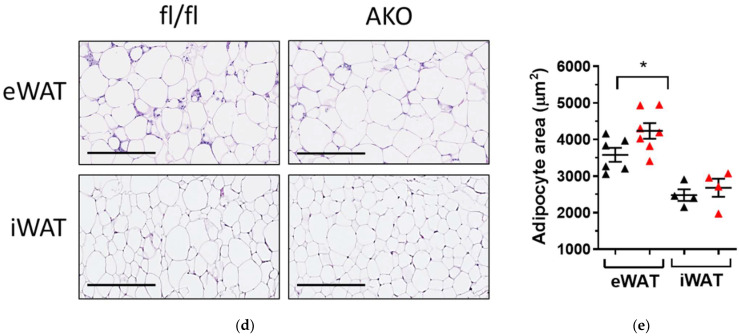
Mice with adipocyte-specific LAMA4 deletion exhibit increased eWAT adipocyte area when maintained on a chow diet. Male *Lama4*^fl/fl^ (fl/fl) and *Lama4*^AKO^ (AKO) mice were provided a chow diet from weaning until 24 weeks of age; body weight was measured weekly and body composition was measured via NMR every other week. (**a**) Knockdown of *Lama4* was confirmed in eWAT and iWAT; heart is shown as a positive control (*n* = 3–4 per genotype). Black bars represent *Lama4*^fl/fl^ mice and red bars represent *Lama4*^AKO^ mice. (**b**) There were no differences in body weight between *Lama4*^fl/fl^ (black) and *Lama4*^AKO^ mice (red) (*n* = 16–18 per genotype). (**c**) Adiposity did not differ between *Lama4*^fl/fl^ (black) and *Lama4*^AKO^ mice (red) (*n* = 16–18 per genotype). (**d**) Representative images of H&E-stained eWAT and iWAT (*n* = 4–6 per group). (**e**) Adipocyte area is significantly increased in eWAT, but not in iWAT, of chow-fed *Lama4*^AKO^ mice (*n* = 4–7 per genotype per tissue). Black triangles represent *Lama4*^fl/fl^ mice and red triangles represent *Lama4*^AKO^ mice. * *p* < 0.05 and ** *p* < 0.01 between genotypes. Scale bars = 250 μm.

Whole-body LAMA4 deletion is associated with decreased fat mass in mice, while obese mice and humans with obesity exhibit increased adipose tissue levels of LAMA4 [11,12]. Here, we investigated whether the loss of adipocyte LAMA4 contributed to alterations in body mass or body composition. We first examined various tissues to confirm *Lama4* knockdown in fat depots from our *Lama4*^AKO^ mice. We observed a knockdown of approximately 50–60% in both eWAT and iWAT of *Lama4*^AKO^ mice when compared to control mice (Figure 3a). Because *Lama4* is expressed in blood vessels and immune cells, we did not expect to observe a complete loss of *Lama4* in adipose tissue. The results we observed, in this case, are consistent with those from whole fat tissue of other adipocyte-specific models we have generated [14,17]. Using control (*Lama4*^fl/fl^) and *Lama4*^AKO^ mice under normal diet conditions, we noted no significant differences in total body mass (Figure 3b), fat mass, or lean mass (Figure 3c) between genotypes. Notably, the fat mass began to diverge toward study completion (Figure 3c). Though both genotypes had similar adiposity, the *Lama4*^AKO^ mice had significantly larger eWAT adipocytes than those of the control mice (Figure 3d,e). No changes in adipocyte size were observed in iWAT.

### 3.3. Adipocyte-Specific LAMA4 Deletion Does Not Affect Glucose Tolerance under Normal Diet Conditions

**Figure 4:**
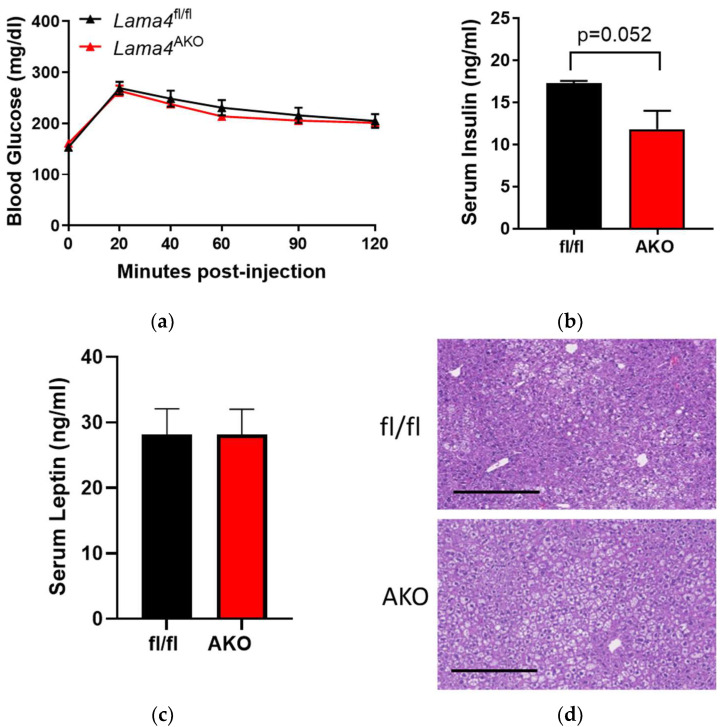
Glucose tolerance is unchanged and postprandial insulin levels are decreased in chow-fed mice with adipocyte-specific LAMA4 deletion. Male *Lama4*^fl/fl^ and *Lama4*^AKO^ mice were provided a chow diet from weaning until 24 weeks of age. (**a**) Intraperitoneal glucose tolerance tests were performed after 18 weeks on diet (*n* = 15–16 mice per genotype after a 4-h fast). Serum (**b**) insulin levels and (**c**) leptin levels in fed mice (*n* = 6 per genotype) were measured by ELISA. (**d**) Representative images of H&E-stained liver sections (*n* = 5–7 per group), demonstrate no differences in hepatic lipid accumulation. Scale bars = 250 μm.

Chow-fed mice with whole-body deletion of LAMA4 are more insulin sensitive and glucose-tolerant than their control counterparts [12]. We examined glucose tolerance, serum insulin, and serum leptin levels in our chow-fed *Lama4^f^*^l/fl^ and *Lama4*^AKO^ mice. Glucose tolerance testing conducted after 18 weeks of feeding revealed no differences between chow-fed *Lama4*^fl/fl^ and *Lama4*^AKO^ mice in terms of the systemic response to a glucose bolus (Figure 4a). Interestingly, however, postprandial insulin levels were lower in *Lama4*^AKO^ mice (*p* = 0.052) when compared to controls at 24 weeks old (Figure 4b). There were also no differences in serum leptin levels between groups (Figure 4c). Histologic evaluation of liver tissue revealed no apparent differences in liver triglyceride content (Figure 4d). 

### 3.4. Adiposity Is Increased in Lama4^AKO^ Mice Fed a High-Fat Diet (HFD)

**Figure 5:**
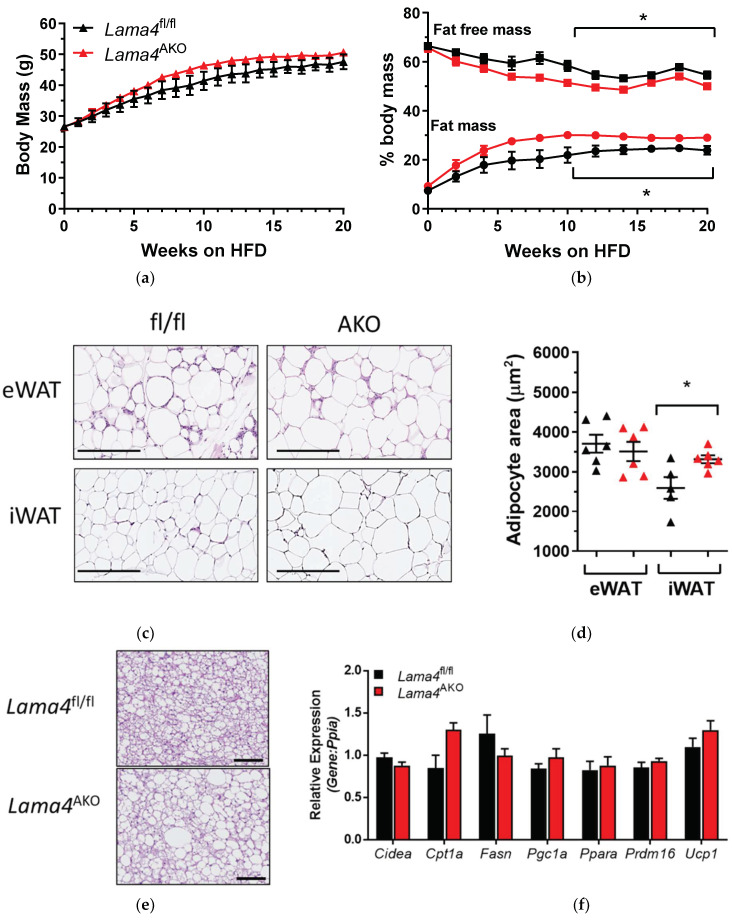
Mice with adipocyte-specific LAMA4 deletion have greater adiposity than control mice when challenged with HFD. Male *Lama4*^fl/fl^ (fl/fl) and *Lama4*^AKO^ (AKO) mice were provided HFD from 6–26 weeks of age; body weight was measured weekly and body composition was measured via NMR every other week. (**a**) There are no differences in body weight between *Lama4*^fl/fl^ (black) and *Lama4*^AKO^ mice (red) (*n* = 16–18 per genotype). (**b**) Adiposity is significantly greater and lean mass is significantly lower (*n* = 16–18 per genotype) in HFD-fed *Lama4*^AKO^ mice (red) when compared to floxed controls (black). (**c**) Representative images of H&E-stained eWAT and iWAT (*n* = 4–7 per genotype). (**d**) Adipocyte area is increased in iWAT, but not in eWAT, of HFD-fed *Lama4*^AKO^ mice (*n* = 4–7 per genotype). Black triangles represent *Lama4*^fl/fl^ mice and red triangles represent *Lama4*^AKO^ mice. (**e**) Larger lipid droplets are present in the BAT of HFD-fed *Lama4*^AKO^ mice (*n* = 4–6 per group), but these morphological changes do not translate to altered BAT gene expression (**f**). * *p* < 0.05 between genotypes. Scale bars = 250 μm.

Adipose tissue LAMA4 levels are increased in human and murine obesity [11], though the consequences of this are unclear. When provided a HFD as an obesogenic stimulus, mice with whole-body LAMA4 deletion exhibit lower fat mass and preservation of insulin sensitivity compared to control mice [12]. In contrast, we noted that although total body mass did not differ between HFD-fed *Lama4^f^*^l/fl^ and *Lama4*^AKO^ mice (Figure 5a), the knockout mice had significantly higher fat mass and significantly lower lean mass (Figure 5b) than the control mice. In contrast to the chow-fed *Lama4*^AKO^ mice, the iWAT of HFD-fed *Lama4*^AKO^ mice contained significantly larger adipocytes (Figure 5c,d). Interestingly, the BAT of HFD-fed *Lama4*^AKO^ mice contained larger lipid droplets (Figure 5e), but the expression of key BAT-associated genes was not altered between genotypes (Figure 5f).

### 3.5. Adipocyte-Specific LAMA4 Deletion Improves Glucose Tolerance in Diet-Induced Obesity

**Figure 6:**
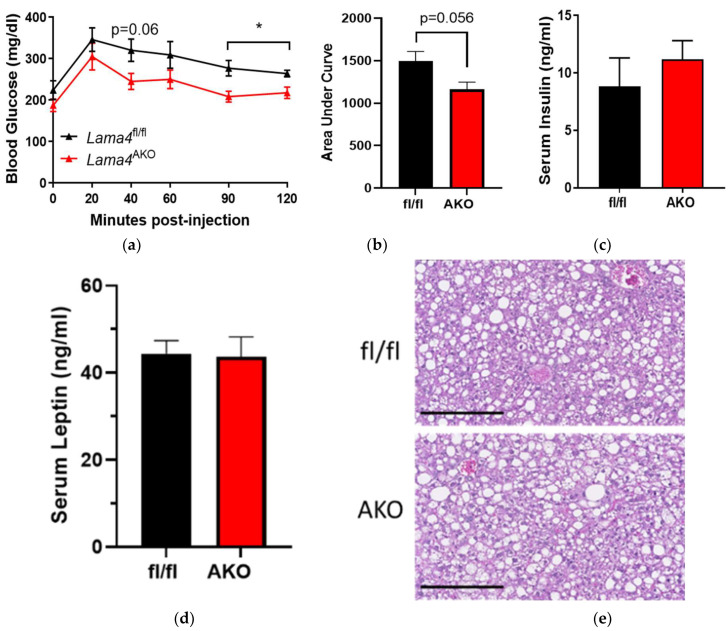
Glucose tolerance is improved, and postprandial insulin levels are unchanged in HFD-fed mice with adipocyte-specific LAMA4 deletion. Male *Lama4*^fl/fl^ and *Lama4*^AKO^ mice were provided HFD from 6–26 weeks of age. (**a**) Intraperitoneal glucose tolerance tests were performed after 18 weeks on diet (*n* = 15–16 mice per genotype after a 4-h fast). (**b**) The area under curve calculated from (**a**). (**c**) Insulin levels and (**d**) leptin levels in serum from fed mice (*n* = 6 per genotype) were measured by ELISA. (**e**) Representative images of H&E-stained liver sections (*n* = 5–7 per group). * *p* < 0.05 between genotypes. Scale bars = 250 μm.

HFD-fed *Lama4*^AKO^ mice, despite having greater adiposity, are more glucose-tolerant than their control counterparts (Figure 6a,b). Further, these mice exhibit no differences in postprandial insulin levels (Figure 6c), leptin levels (Figure 6d), or hepatic lipid accumulation (Figure 6e). Our glucose tolerance findings coincide with those reported in *Lama4^−/−^* mice. However, the striking differences in adiposity between the models suggest that the underlying mechanisms for the improved glucose tolerance may not be the same; this will require further study.

### 3.6. RNA-Seq Identifies Distinct Transcriptomic Differences in eWAT in Response to Diet and Genotype

We observed during the microscopic evaluation of adipose tissue depots that eWAT from chow- and HFD-fed *Lama4*^AKO^ mice had less inflammatory infiltrate than eWAT from control animals. Given the influence of inflammation on many functions of adipose tissue, we conducted bulk RNA sequencing on eWAT from chow- and HFD-fed mice of each genotype to better assess genes and pathways affected by *Lama4* deletion. 

**Figure 7:**
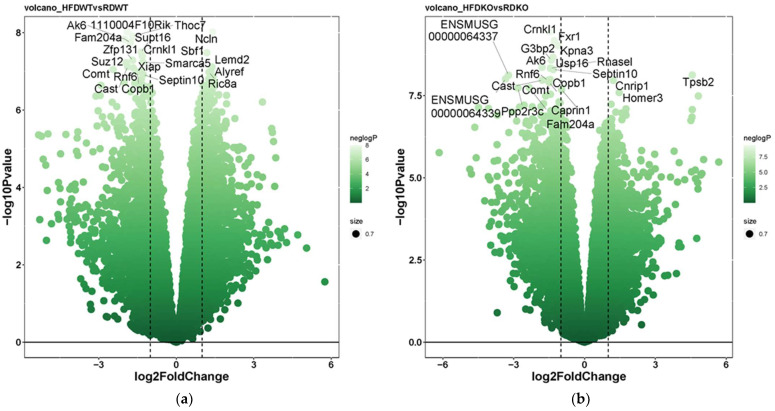

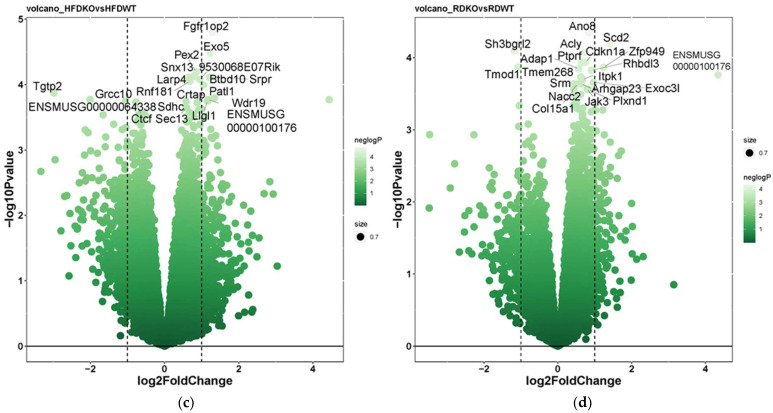
Volcano plots depicting differential eWAT expression patterns across diet (chow or HFD) or genotype (*Lama4*^fl/fl^ mice or *Lama4*^AKO^) comparisons. (**a**) High-fat vs. chow diet comparison in *Lama4*^fl/fl^ mice; (**b**) High-fat vs. chow diet comparison in *Lama4*^AKO^ mice; (**c**) *Lama4*^fl/fl^ mice vs. *Lama4*^AKO^ mice on a high-fat diet; (**d**) *Lama4*^fl/fl^ mice vs. *Lama4*^AKO^ mice on chow diet. RD = chow diet, HFD = high-fat diet, WT = *Lama4*^fl/fl^ and KO = *Lama4*^AKO^. The top 20 differentially expressed genes are annotated in each volcano plot, with full gene names appearing in Appendix B. Dashed lines represent the significance cutoffs for identifying genes as significantly regulated.

Our RNA-Seq analysis identified distinct differences in gene expression in response to diet and genotype. A total of 1800 genes were differentially expressed between chow- and HFD-fed *Lama4*^fl/fl^ mice (absolute fold-change ≥ 1.5-fold, nominal *p*-value ≤ 0.001, maximum average group expression ≥ 2 log CPM). Under the same conditions, 2218 genes were differentially expressed between chow- and HFD-fed *Lama4*^AKO^ mice. When comparing HFD-fed *Lama4*^fl/fl^ mice to HFD-fed *Lama4*^AKO^ mice, 79 genes were differentially expressed, whereas only 31 genes satisfied the conditions for differential expression between chow-fed *Lama*4^fl/fl^ and *Lama*4^AKO^ mice. Differential expression patterns across these four comparisons are depicted via volcano plots in Figure 7a–d, with the top 20 differentially expressed genes annotated in each plot (full gene names appear in Appendix B).

**Figure 8:**
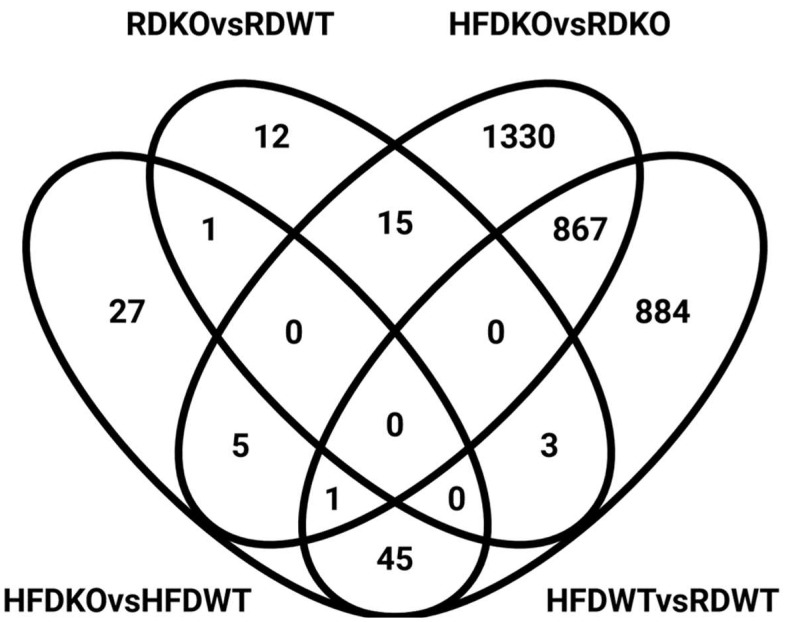
Venn diagram depicting the overlap among differentially expressed genes from each diet (chow or HFD) or genotype (*Lama4*^fl/fl^ mice or *Lama4*^AKO^) comparison. RD = chow diet, HFD = high-fat diet, WT = *Lama4*^fl/fl^ and KO = *Lama4*^AKO^.

**Figure 9:**
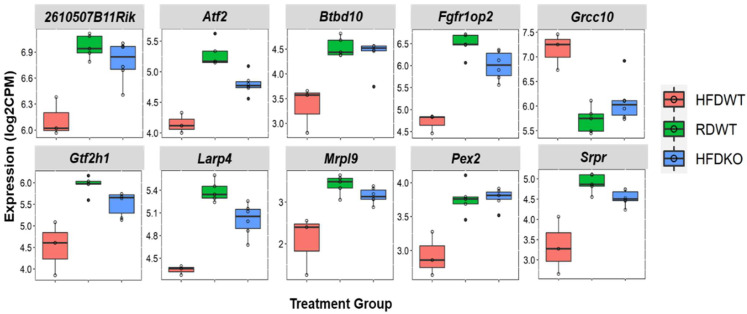
Boxplots of top ten genes differentially expressed in common between HFD-fed *Lama4*^fl/fl^ and *Lama4*^AKO^ mice, as well as between HFD and chow-fed *Lama4*^fl/fl^ mice. log2CPM = log 2 counts per million, RD = chow diet, HFD = high-fat diet, WT = *Lama4*^fl/fl^ and KO = *Lama4*^AKO^. *Atf2* = activating transcription factor 2, *Btbd10* = BTB domain containing 10, *Fgfr1op2* = FGFR1 oncogene partner 2, *Grcc10* = gene-rich cluster, C10 gene, *Gtf2h1* = general transcription factor IIH subunit 1, *Larp4* = La ribonucleoprotein 4, *Mrpl9* = mitochondrial ribosomal protein L9, *Pex2* = peroxisomal biogenesis factor 2, *Srpr* = signal recognition particle receptor subunit alpha.

We next investigated the extent of overlap among the differentially expressed genes from each comparison (Figure 8) and found 887 genes to be regulated in common in response to diet between *Lama4*^fl/fl^ and *Lama4*^AKO^ mice. In contrast, there was almost no overlap between *Lama4*^fl/fl^ and *Lama4*^AKO^ mice on chow or HFD. A group of 45 genes was found to be differentially expressed in common between HFD-fed *Lama4*^fl/fl^ and *Lama4*^AKO^ mice, as well as between HFD and chow-fed control mice (Figure 8). These genes spanned various biological functions including peroxisomal and ribosomal function, protein targeting, protein regulation, RNA regulation, and DNA damage (Supplementary Table S1). A detailed analysis of the direction of expression of these 45 genes showed both HFD-fed *Lama4*^AKO^ mice and chow-fed *Lama4*^fl/fl^ mice to display similar changes in gene expression compared to HFD-fed *Lama4*^fl/fl^ mice, suggesting that adipocyte LAMA4 loss under HFD conditions mimics a transcriptomic phenotype similar to that of chow-fed control animals. The expression changes of the top 10 genes in this category (selection based on averaged *p*-values across the comparisons) are shown in Figure 9 (expression profiles of all 45 genes shown in Supplementary Figure S4a,b).

**Figure 10:**
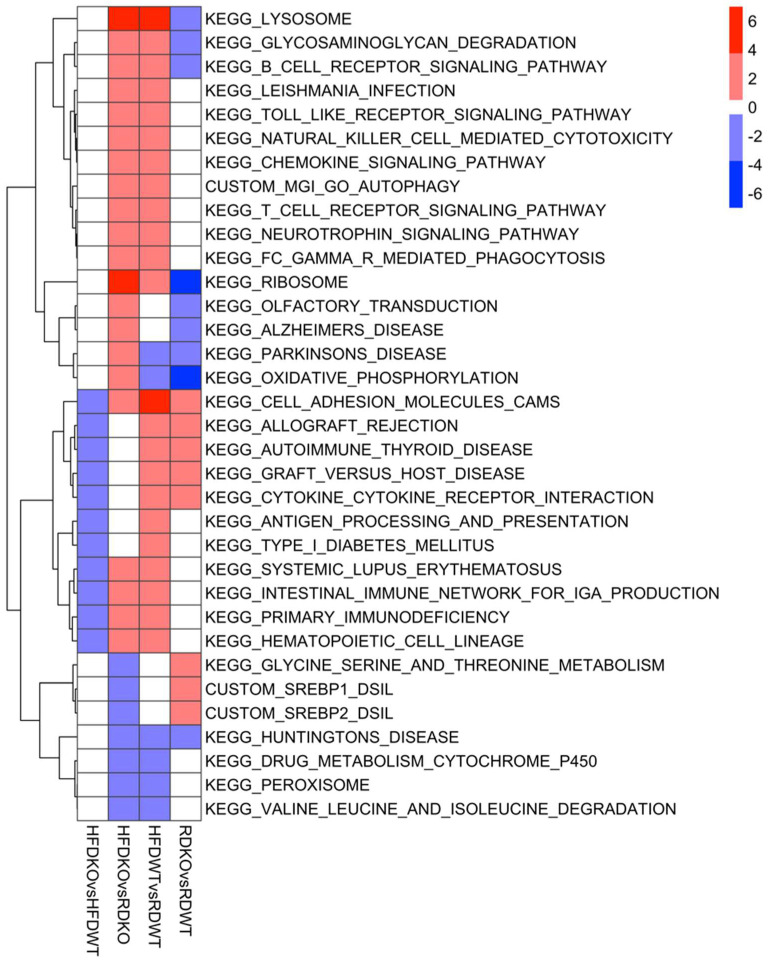
Heatmap summarizing significantly up—(red) or down—(blue) regulated as determined by GSEA (FDR ≤ 0.05). Pathways showing significant regulation (FDR ≤ 0.05) in two or more diet or genotype comparisons are shown. RD = chow diet, HFD = high-fat diet, WT = *Lama4*^fl/fl^ (control) mice, KO = *Lama4*^AKO^ mice. For the HFDKO vs. HFDWT column, pathway directionality reported is for the HFDWT group (i.e., red = upregulated in HFDWT vs. HFDKO). For the HFDKO vs. RDKO column, directionality is reported for the RDKO group. For the HFDWT vs. RDWT and RDKO vs. RDWT columns, directionality is reported for the RDWT group.

**Figure 11:**
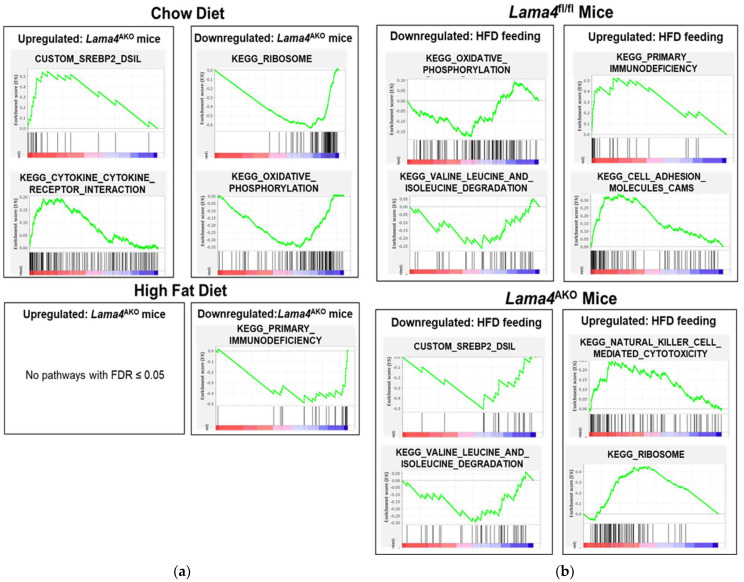
Enrichment plots of selected pathways significantly up-or down-regulated (FDR ≤ 0.05) in two or more diet or genotype comparisons as determined by GSEA and summarized in Figure 10. (**a**) Genotype comparisons revealed that sterol and immune pathways were upregulated, while ribosomal and oxidative phosphorylation pathways were downregulated, in chow-fed *Lama4*^AKO^ mice when compared to chow-fed controls. Conversely, no pathways were upregulated, while immune pathways were downregulated in HFD-fed *Lama4*^AKO^ mice when compared to controls. (**b**) Diet comparisons revealed significant downregulation of metabolism-related pathways and upregulation of inflammatory pathways upon HFD feeding compared to chow feeding in *Lama4*^fl/fl^ mice. A similar pattern of pathway changes was also observed in *Lama4*^AKO^ mice with HFD feeding.

We also conducted gene-set enrichment analysis to identify the up- and down-regulation of specific pathways based on the expression changes of their constituent genes. A summary of the pathways significantly altered (FDR ≤ 0.05) in two or more comparisons is shown in Figure 10, and enrichment plots of selected pathways are shown in Figure 11.

When comparing across genotypes, chow-diet feeding produced a significant upregulation in a subset of immune/inflammation pathways (e.g., cytokine-cytokine receptor interaction) and pathways related to sterol biosynthesis (sterol regulatory element-binding transcription factor 1 (SREBF1)- and SREBF2-regulated) in *Lama4*^AKO^ mice compared to *Lama4*^fl/fl^ mice, whereas pathways related to ribosome function and oxidative phosphorylation were significantly downregulated in *Lama4*^AKO^ mice (Figure 11a). In contrast, HFD feeding resulted in a significant downregulation of several immune/inflammatory signaling (e.g., primary immunodeficiency) pathways in *Lama4*^AKO^ compared to *Lama4*^fl/fl^ mice, whereas no pathways were significantly upregulated in *Lama4*^AKO^ mice (Figure 11a).

When comparing across diets, *Lama4*^fl/fl^ mice showed significant downregulation of a subset of metabolism-related pathways upon HFD feeding compared to chow feeding (branched-chain amino acid metabolism, oxidative phosphorylation, etc.; Figure 11b, top), whereas several immune/inflammatory pathways were upregulated in the HFD-fed group (primary immunodeficiency, etc.). A similar pattern of pathway changes was also observed in *Lama4*^AKO^ mice with downregulation of metabolism-related and upregulation of immuno-inflammatory signaling pathways upon HFD feeding (Figure 11b, bottom).

## 4. Discussion

Laminin α4 (LAMA4) is the primary alpha subunit found in the laminins comprising the basement membranes of human and mouse adipocytes. Possible roles for LAMA4 in metabolism have been uncovered by extensive profiling of the whole-body LAMA4 knockout mouse, with *Lama4^−^*^/−^ mice having lower adiposity, higher energy expenditure, and greater insulin sensitivity than their wild-type counterparts [4,11,12]. However, the LAMA4 subunit is important in cardiovascular health, and cardiovascular defects reported in *Lama4^−/−^* mice [9,10] may confound the interpretation of metabolic outcomes. In this study, we tested the hypothesis that deletion of LAMA4 specifically in mature adipocytes would affect adipose tissue in a manner that contrasted with that of whole-body LAMA4 deletion. This work resulted in several key findings: (1) laminin α4 is highly expressed in mouse and human adipocytes; (2) in the diet-induced obese condition, the *Lama4*^AKO^ mouse remains slightly more glucose tolerant despite being significantly fatter; 3) transcriptionally, eWAT from the HFD-fed *Lama4*^AKO^ mouse resembles that of the chow-fed *Lama4*^fl/fl^ mouse, suggesting a protective effect of adipocyte LAMA4 deletion.

Under normal physiological conditions (chow-diet feeding), body weight and body composition do not differ between *Lama4*^fl/fl^ and *Lama4*^AKO^ mice, though eWAT adipocyte size is significantly increased in *Lama4*^AKO^ mice. These findings directly contrast with the significant decreases in body weights and eWAT masses reported in *Lama4^−/−^* mice [12] and may be partly explained by the potential vascular effects of the whole-body knockout. Adipose tissue development and expansion require a functional vascular network that can also expand to maintain adequate blood flow [24,25,26]. Indeed, eWAT develops postnatally, with vascular expansion preceding adipocyte formation [26], so defective microvessel expansion would certainly affect this process. Interestingly, both *Lama4^−/−^* and *Lama4*^AKO^ mice had larger eWAT adipocytes, suggesting that one of the adipocyte-autonomous effects of LAMA4 loss is an increase in cell size.

In terms of transcriptomic analyses of eWAT, few genes were significantly regulated between chow-fed *Lama4*^fl/fl^ and *Lama4*^AKO^ mice. Notably, at the gene level, *Col15a1* and *Plxnd1* were upregulated in *Lama4*^AKO^ mice. Collagen XV is associated with many basement membranes [5,6], while plexin D1 can physically associate with LAMA4 [27] and has a regulatory role in visceral adipose tissue lipid storage [28,29,30]. At the pathway level, oxidative phosphorylation and ribosomal pathways were downregulated in *Lama4*^AKO^ mice, while cytokine-receptor and sterol pathways were upregulated. When taken together, these data suggest that adipocyte LAMA4 loss results in changes in eWAT adipocyte membrane composition that are permissive to an increase in cell size and downregulation of lipid metabolism, leading to increased lipid storage.

The most striking findings came from our analysis of *Lama4*^fl/fl^ and *Lama4*^AKO^ mice challenged with an obesogenic stimulus (in this case, HFD feeding). Though body weight did not differ between HFD-fed *Lama4*^fl/fl^ and *Lama4*^AKO^ mice, the *Lama4*^AKO^ mice had significantly more adipose tissue and significantly less lean mass. Unlike our observations in chow-fed *Lama4*^AKO^ mice, eWAT adipocyte area was unchanged in HFD-fed *Lama4*^AKO^ mice. However, total adiposity and iWAT adipocyte area were increased and accompanied by apparent preservation of glucose tolerance and no change in leptin levels. These findings are again in contrast to those from *Lama4^−/−^* mice, as the adipocyte-specific knockouts are resistant to HFD-induced weight gain. Further, HFD-fed *Lama4^−/−^* mice exhibit iWAT beiging and increased energy expenditure but no changes in BAT morphology or gene expression [4]. Like *Lama4^−/−^* mice, our HFD-fed *Lama4*^AKO^ mice have no changes in BAT gene expression. As noted above, the expansion of adipose tissue, whether developmentally or in response to dietary cues, requires an intact and functional vascular system and the documented impairment in *Lama4*-null vessels may be a factor in the differences between our model and the *Lama4^−/−^* mouse. Further, our transcriptomic analyses revealed significant similarities between eWAT from HFD-fed *Lama4*^AKO^ and chow-fed *Lama4*^fl/fl^ mice, suggesting an overall protective function of adipocyte LAMA4 loss in response to an obesogenic challenge.

There are several limitations to this study that should be considered. First, we did not perform detailed metabolic phenotypic analyses of our *Lama4*^fl/fl^ and *Lama4*^AKO^ mice. Thus, we are unable to determine whether HFD-fed *Lama4*^AKO^ mice have more adipose tissue in response to a decrease in energy expenditure or activity, or due to an increase in food intake or both. Given the morphological changes in both white and brown fat depots in our knockouts, it will be critical to assess metabolic phenotype in future studies. The small group sizes in our HFD study also limit the conclusions we can draw from these analyses. Moreover, though our transcriptomic and microscopic analyses revealed a decreased inflammatory burden in the eWAT of HFD-fed *Lama4*^AKO^ mice, we did not determine which immune cell populations are affected. Indeed, deletion of LAMA4 in fibroblastic reticular cells of the lymph node affects Treg and dendritic cell populations [31], so it will be important in the future to examine how immune cell populations are regulated by adipocyte LAMA4 deletion. Lastly, we performed our initial analyses on male mice only at a single age. Given the known sex-based and age-related differences in adipose tissue function and immune cell populations, it will be important to examine both sexes at various ages in future studies.

Our findings, in combination with those from the *Lama4^−/−^* mouse, reveal a significant role for LAMA4 in adipose tissue physiology while also highlighting the need for more detailed mechanistic studies. Future studies will focus on the mechanisms surrounding how adipocyte laminins may influence intracellular signaling cascades, immune cell activity, lipid storage, and overall metabolic health. Additional studies will examine how other adipocyte basement membrane components interact with laminins to exert these effects.

## Data Availability

Raw and processed RNA-Seq data are deposited in the GEO database (accession number GEO: GSE179138).

## Supplementary Materials

The following supporting information can be downloaded at: https://www.mdpi.com/article/10.3390/biomedicines10092077/s1, Figure S1: Principal Components Analysis used to identify outliers. Figure S2: *LAMA4* expression in human white adipose tissue cells; Figure S3: *Lama4* expression in mouse white adipose tissue cells; Figure S4a,b: Boxplots of top genes differentially expressed in common between HFD-fed *Lama4*^fl/fl^ and *Lama4*^AKO^ mice as well as between HFD and chow-fed *Lama4*^fl/fl^ mice; Table S1: Functions of genes reported as differentially expressed in Figure 8, Figure 9 and Figure S4a,b.

## Appendix A

### Primers Used to Validate Recombination

For verifying recombination in ES cells, primers upstream of the 5′ recombination arm and downstream of the 3′ recombination arm were each paired with an appropriate primer located within the Neo portion of the vector. Approximate primer locations are labeled in Figure 1 of the main text.

5′ arm recombinant pair: GF1 5′-CTCATTAACTTCCTTACTAGGACCTAAGC-3′,

NR1 5′-CTTGGCTGGACGTAAACTCCTCTTCAGA-3′.

Product size = 6.3 kb

3′arm recombinant pair: NF1 5′-GCTCTATGGCTTCTGAGGCGGAAAGAA-3′,

GR1 5′-AAGCTCAAAGAGGAAGCGTCTG-3′.

Product size = 5 kb

## Appendix B

### Top Differentially Expressed eWAT Genes across Diet or Genotype Comparisons

Full gene names for the top 20 genes expressed in each comparison depicted in Figure 7 appear in alphabetical order, below. Names for genes appearing in more than one comparison are listed where they first appear.

Figure 7a: *Ak6* = adenylate kinase 6, *Alyref* = Aly/REF export factor, *Cast* = calpastatin, *Copb1* = COPI coat complex subunit beta 1, *Comt* = catechol-O-methyltransferase, *Crnkl1* = crooked neck pre-mRNA splicing factor 1, *Fam204a* = family with sequence similarity 204 member A, *Lemd2* = LEM domain nuclear envelope protein 2, *Ncln* = nicalin, *Ric8a* = RIC8 guanine nucleotide exchange factor A, *Rnf6* = ring finger protein 6, *Sbf1* = SET binding factor 1, *Septin10* = Septin 10, *Smarca5* = SWI/SNF-related matrix-associated actin-dependent regulator of chromatin subfamily A member 5, *Supt6* = SPT6, histone chaperone and transcription elongation factor, *Suz12* = SUZ12 polycomb repressive complex 2 subunit, *Thoc7* = THO complex subunit 7, *Xiap* = X-linked inhibitor of apoptosis protein, *Zfp131* = zinc finger protein 131.

Figure 7b: *Caprin1* = cytoplasmic activation/proliferation-associated protein-1, *Cnrip1* = cannabinoid receptor interacting protein 1, *Fxr1* = fragile X mental retardation syndrome-related protein 1, *G3bp2* = G3BP stress granule assembly factor 2, *Homer3* = homer scaffold protein 3, *Kpna3* = karyopherin subunit alpha 3, *Ppp2r3c* = protein phosphatase 2 regulatory subunit B″ gamma, *Rnasel* = ribonuclease L, *Tpsb2* = tryptase beta 2, *Usp16* = ubiquitin specific peptidase 16.

Figure 7c: *Btbd10* = BTB domain containing 10, *Crtap* = cartilage associated protein, *Ctcf* = CCCTC-binding factor, *Exo5* = exonuclease 5, *Fgfr1op2* = fibroblast growth factor receptor 1 oncogene partner 2, *Grcc10* = gene rich cluster, C10 gene, *Larp4* = La ribonucleoprotein 4, *Llgl1* = LLGL scribble cell polarity complex component 1, *Patl1* = PAT1 homolog 1, processing body mRNA decay factor, *Pex2* = peroxisomal biogenesis factor 2, *Rnf181* = ring finger protein 181, *Sdhc* = succinate dehydrogenase complex subunit C, *Sec13* = SEC13 homolog, nuclear pore and COPII coat complex component, *Snx13* = sorting nexin 13, *Srpr* = signal recognition particle receptor subunit alpha, *Tgtp2* = T cell specific GTPase 2, *Wdr19* = WD repeat domain 19.

Figure 7d: *Adap1* = Arf-GAP with dual PH domain-containing protein 1, *Ano8* = anoctamin 8, *Arhgap23* = Rho GTPase activating protein 23, *Cdkn1a* = cyclin-dependent kinase inhibitor 1a, *Col15a1* = collagen alpha-1(XV) chain, *Exoc3l* = exocyst complex component 3-like, *Itpk1* = inositol tetrakisphosphate 1-kinase 1, *Jak3* = janus kinase 3, *Nacc2* = nucleus accumbens-associated protein 2, *Plxnd1* = plexin D1, *Ptprf* = receptor-type tyrosine-protein phosphatase F, *Rhbdl3* = rhomboid-like 3, *Scd2* = stearoyl-CoA desaturase 2, *Scly* = selenocysteine lyase, *Sh3bgrl2* = SH3 domain binding glutamic acid-rich protein like 2, *Srm* = spermidine synthase, *Tmem268* = transmembrane protein 268, *Tmod1* = tropomodulin 1, *Zfp949* = zinc finger protein 949.
