# Supplementary material for: Adipocyte-Specific Laminin Alpha 4 Deletion Preserves Adipose Tissue Health despite Increasing Adiposity
Source: Biomedicines. 2022 Aug 25;10(9):2077. doi: 10.3390/biomedicines10092077 (PMC9495590; doi:10.3390/biomedicines10092077)
Supplement: Supplementary file 1 [file biomedicines-10-02077-s001.zip › biomedicines-1862041-supplementary.pdf]

Supplementary Figure S1

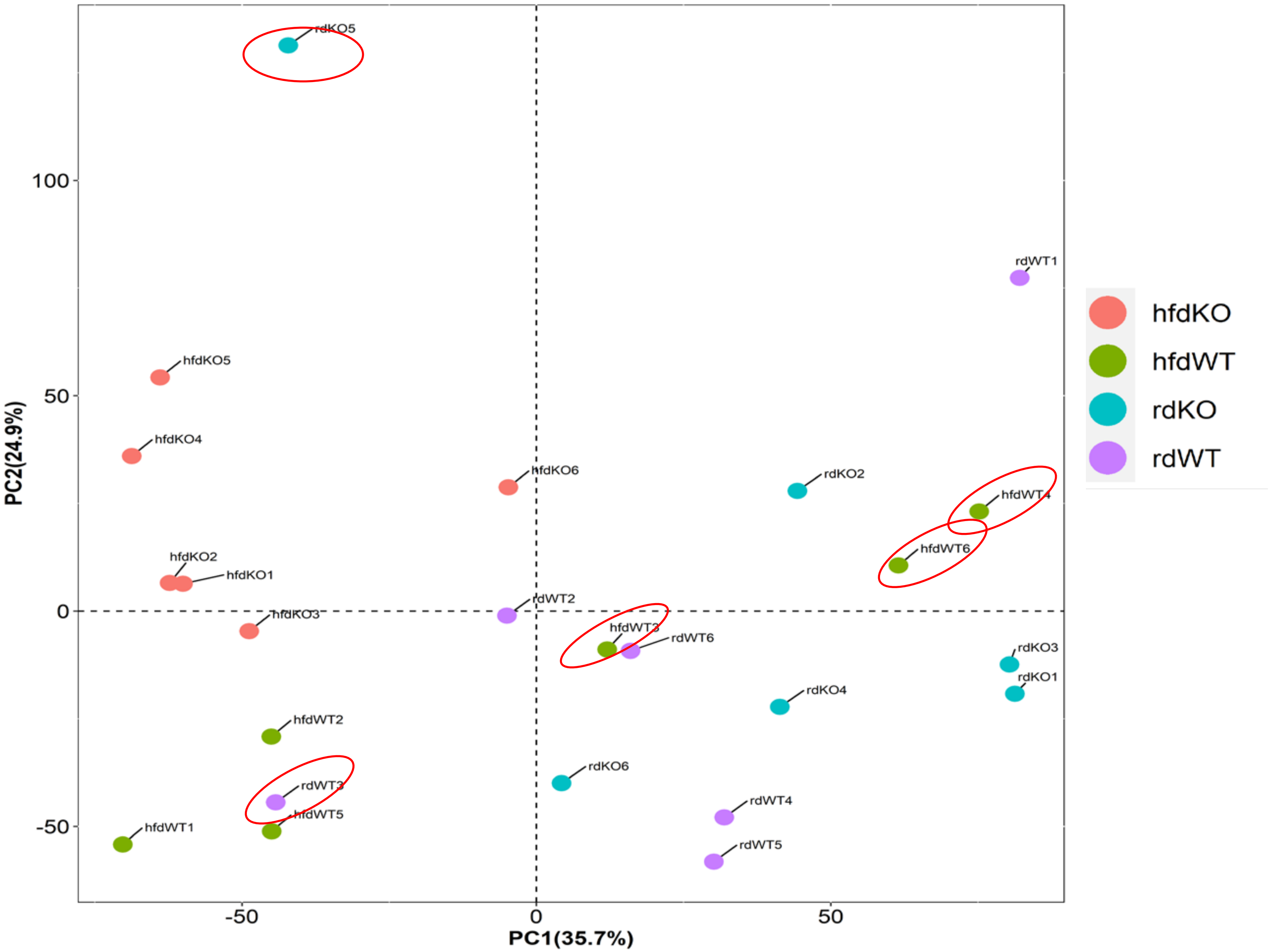

Supplementary Figure S2

Human White Adipose Tissue

Nuclei Clustering | ***LAMA4* Expression**

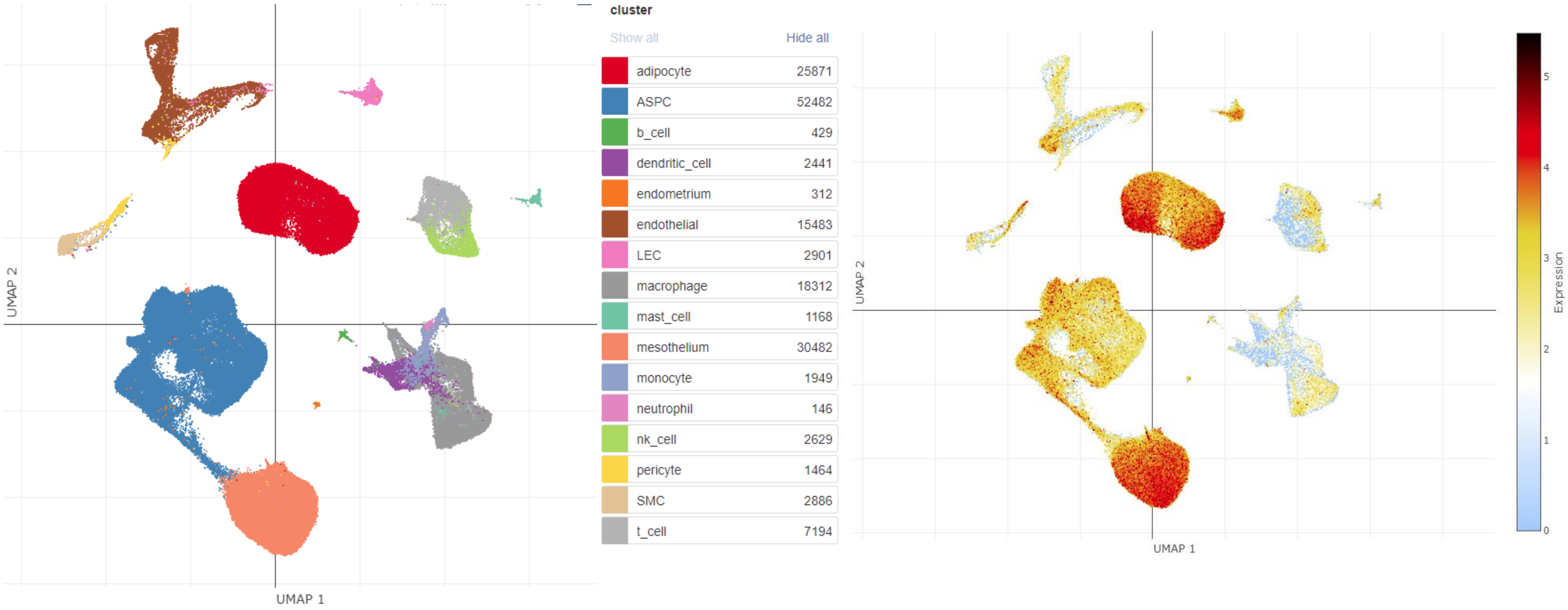

Supplementary Figure S3

Mouse White Adipose Tissue

Nuclei Clustering | *Lama4* Expression

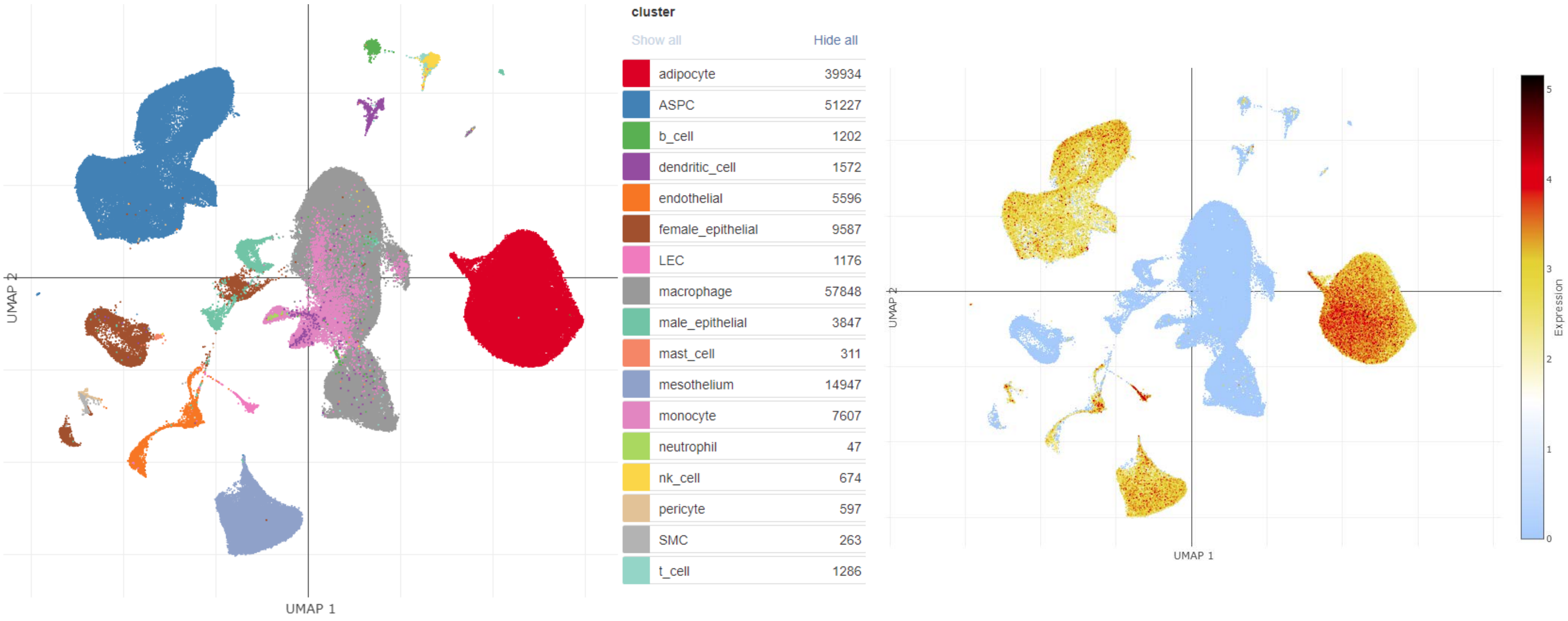

Supplementary Figure S4a

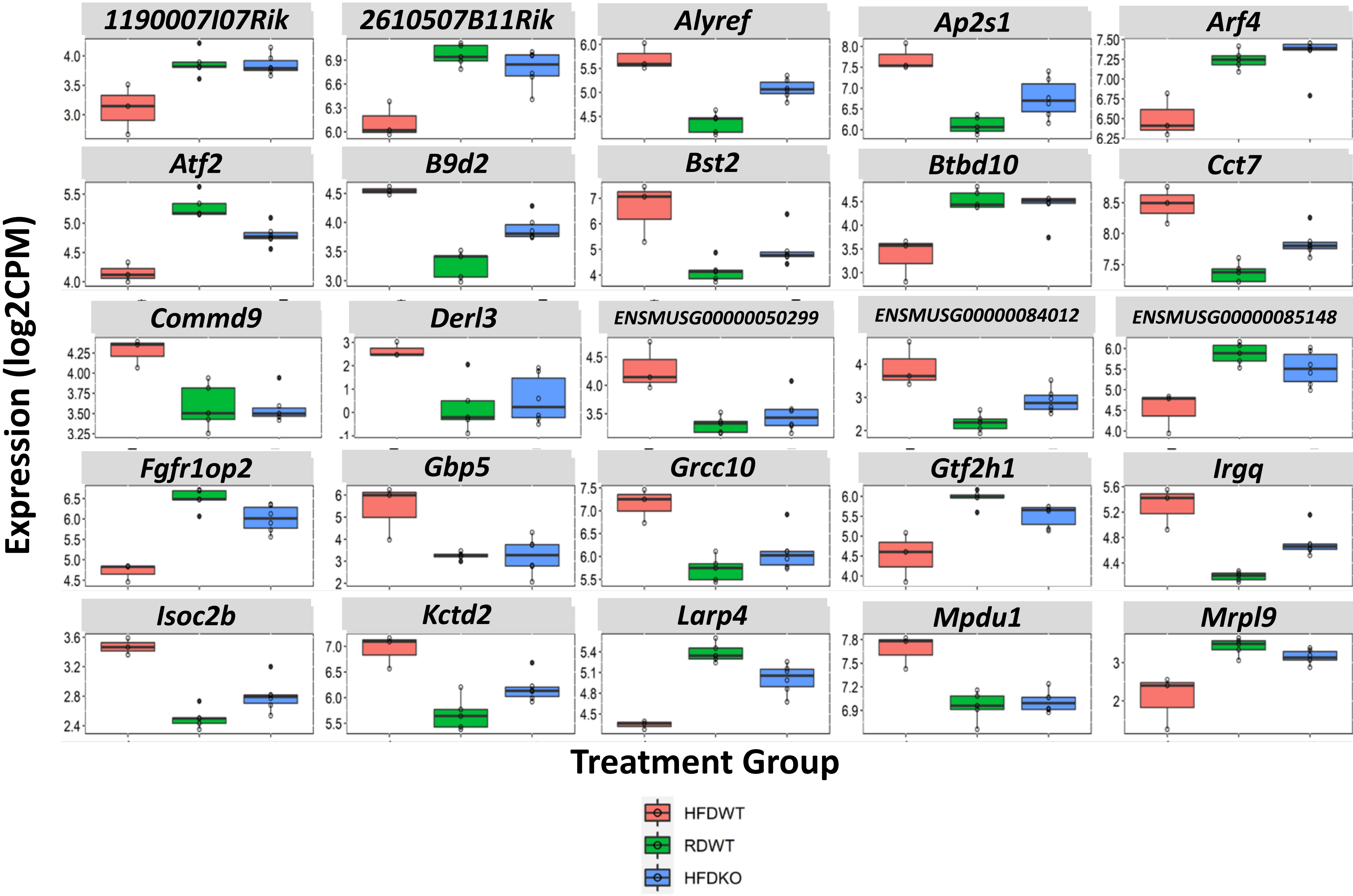

Supplementary Figure S4b

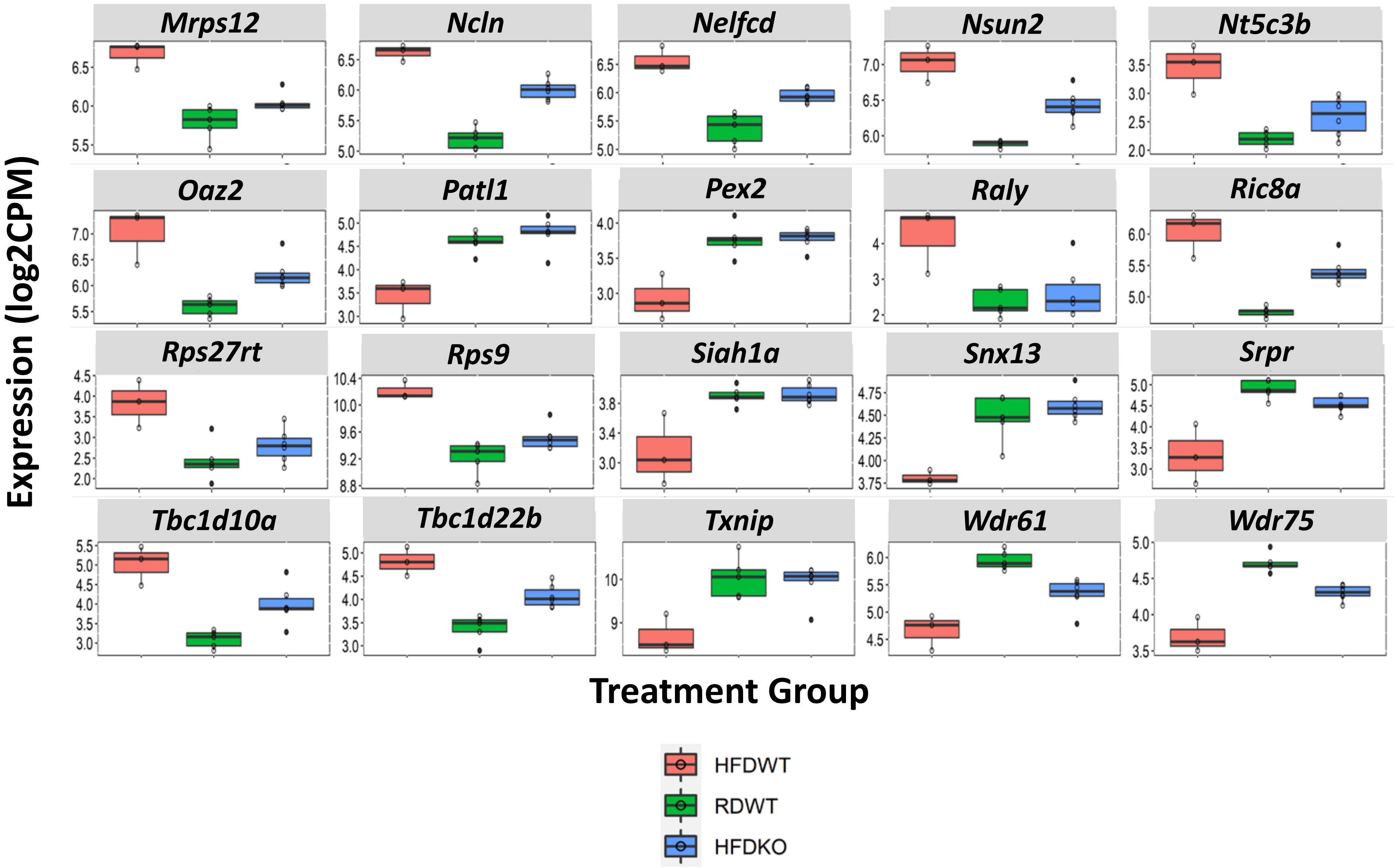

Supplementary Table S1

gene	Function	peroxisome	ribosome	protein targeting	DNA damage	RNA regulation	G-protein regulation	protein regulation
Fgfr1op2	wound healing							
Larp4	regulation of mRNA translation, associates with ribosomal subunit							
Srpr	nascent protein targeting to ER							
Btbd10	Akt activation via inhibition of dephosphorylation by PPP2CA							
Pex2	Peroxisome biogenesis							
Grcc10	required for corpus callosum development							
Gtf2h1	general and transcription-coupled nucleotide excision repair of damaged DNA							
2610507B11Rik	unknown function							
Mrpl9	mitochondrial ribosome component, associated with peroxisome biogenesis disorder							
Atf2	transcriptinal activator of genes involved in anti-apoptosis, cell growth and DNA-damage response							
Ncln	ribosome-associated translocon complex component, regulation of protein complex stability							
Tbc1d22b	GTPase activating protein for Rab family proteins, intracellular protein transport							
Mrps12	component of ribosome							
B9d2	regulation of ciliogenesis							
Mpdu1	regulation of lipid-linked oligosaccharides and glycosylphosphatidylinositol synthesis							
Tbc1d10a	GTPase activting protein for Rab27A, retrograde protein transport from endosome to Golgi							
Nsun2	methylation of cytosine to promote TRNA stability and prevent MRNA decay							
Nt5c3b	involved in exonucleolytic catabolism of deadenylated MRNA							
Ric8a	G-alpha protein activator, regulation of GPCR activity including basement membrane organization							
Irgq	GTP binding activity							
Snx13	regulation of G protein signaling, involved in intracellular trafficking							
Alyref	nuclear located molecular chaperone, regulation of BZIP proteins, nuclear export of MRNA							
NA	unknown function							
Rps9	ribosomal protein, component of 40S subunit							
Isoc2b	involved in protein stabilization							
Commd9	involved in sodium ion transport, act upstream of or within cholesterol homeostasis, NFKb regulation							
Cct7	molecular chaperone, part of TCP1 complex, involved in folding of nascent proteins including actin and tubulin							
Derl3	degradation of misfolded glycoproteins in ER, functional component of ERAD							
NA	unknown function							
Kctd2	involved in proteasome mediated protein catabolism							
Txnip	inhibitor of thioredoxin, regulation of cellular redox signaling and oxidative stress and ER stress							
Wdr75	RNA binding, regulatin of ribosomal RNA processing and RNAPOL1 transcription							
Raly	RNA binding protein, transcriptional cofactor of cholesterol biosynthetic genes in liver							
Nelfcd	repressor of RNApol II mediated transcriptional elongation, component of NELF complex							
Ap2s1	clathrin associated, involved in protein transport							
NA	lncRNA, unknown function							
Oaz2	negative regulator of ornithine decarboxylase activity and intracellular polyamine biosynthesis							
Wdr61	component of PAF and SKI complexes, transcriptional regulation including RNA surveillance							
Bst2	antiviral activity via tethering nascent virions to infected cell membranes							
Rps27rt	component of 40S ribosome, regulation of rRNA processing							
Gbp5	component of TRAFAC class GTPases, activator of NLRP3 inflammasome assembly							
Patl1	RNA binding protein involved in deadenylation-dependent decapping of mRNAs leading to their degradation							
Siah1a	E3 ligase mediates ubiquitination and proteosomal degradation of target proteins							
1190007I07Rik	unknown function							
Arf4	GTP-binding protein, activator of cholera toxin, involved in protein trafficking							
